# Nitric oxide and membrane lipid peroxidation in photosynthetic and non-photosynthetic organisms under several stress conditions

**DOI:** 10.3389/fphys.2013.00276

**Published:** 2013-10-17

**Authors:** Andrea Galatro, Paula M. González, Gabriela Malanga, Elizabeth Robello, Natacha E. Piloni, Susana Puntarulo

**Affiliations:** Physical Chemistry, School of Pharmacy and Biochemistry, Institute of Biochemistry and Molecular Medicine, University of Buenos Aires-CONICETBuenos Aires, Argentina

**Keywords:** nitric oxide, lipid peroxidation, photosynthetic organisms, animals, oxidative stress conditions

## Introduction

Oxidative damage to lipids was characterized in terms of the nature of the oxidant, the type of lipid, and the severity of the oxidation (Simontacchi et al., 2011). Even though malondialdehyde detection with the thiobarbituric acid reactive substances test (TBARS) is the most currently used assay for the determination of lipid oxidation, it is unspecific since the reaction can be reproduced by other biological compounds (Simontacchi et al., 2011). On the other hand, electron paramagnetic resonance (EPR) spectroscopy showed the capacity of detecting the presence of the lipid radicals (LR^•^) formed during peroxidation, by yielding unique and stable products with spin traps (Malanga and Puntarulo, 2012). Nitric oxide (NO) is recognized both, as a signaling molecule that regulates many enzyme activities, but as a toxic agent as well. It has been found that NO is able to protect animal and plant cell types from oxidative damage resulting from superoxide (O^−^_2_), hydrogen peroxide (H_2_O_2_) and alkyl peroxides by acting as a terminator of free radical chain reactions (Wink et al., 1995, 1996; Yalowich et al., 1999; Beligni and Lamattina, 2002; Sharpe et al., 2003). Reactive oxygen species (ROS) and reactive nitrogen species (RNS) interact through the reaction of O^−^_2_ with NO, to generate peroxynitrite (ONOO^−^) at a rate close to diffusion. ONOO^−^ acts as both, a nitrating agent and a powerful oxidant capable of modifying proteins (formation of nitrotyrosine), lipids (lipid oxidation, lipid nitration), and nucleic acids (DNA oxidation and DNA nitration) (Gisone et al., 2004).

The purpose of this commentary is to point out that NO complex interactions with other cellular components lead to a wide range of effects depending on the biological system under study and the oxidative stress condition.

## Lipid peroxidation and no in photosynthetic organisms

In cultures of the green algae *Chlorella vulgaris* no significant changes were observed in either of the parameters showed in Table 1, in the stationary phase as compared to the log phase of growth. However, Qian et al. (2009) demonstrated in *Chlorella vulgaris* that, depending on its concentration, NO increased the activity of antioxidant enzymes to protect against the oxidative damage caused by herbicide stress. Data from Simontachi et al. (2004) showed that the NO steady state concentration in homogenates from sorghum embryonic axes reached a maximum at 24–30 h after the starting of imbibition, in coincidence with the initiation of an active germination. The generation of LR^•^ in sorghum axes was drastically increased at 36 h of imbibition from non-detectable values at 24 h, coincidentally with the significant decline in the content of cellular NO (Table 1).

**Table 1:** **Nitric oxide and lipid peroxidation under stress conditions in photosynthetic and non-photosynthetic organisms**.

**Biological system**	**Stress condition**	**Lipid peroxidation**		**NO**	**References**
		**TBARS**	**LR^•^**		
**PHOTOSYNTHETIC ORGANISMS**					
**Intact cells**					
*Chlorella* cells	Development	No change day 12–18	No change day 12–18	No change day 12–18	Malanga and Puntarulo, 1995; Estevez et al., 2001; Estévez and Puntarulo, 2005
**Homogenates**					
Sorghum embryonic axes	Development	nd	Increased from 36 to 48	Decreased from 36 to 48 h	Simontachi et al., 2004
Soybean cotyledons	Senescence	nd	No change day 10–25	Non-detectable at day 25	Jasid et al., 2009
	+SNP	nd	Decrease 58% day 10–25	No change day 10–25	
**Sub-cellular structures**					
Chloroplasts from soybean leaves	+GSNO 250 μM	nd	Decrease 29%	2 μM NO (supplementation)	Jasid et al., 2006
Microsomes from sorghum embryonic axes	+SNP 1 mM	nd	Decrease 43%	Increase 140%	Jasid et al., 2008
**NON-PHOTOSYNTHETIC ORGANISMS**					
**Invertebrates**					
*Nacella magellanica*	Summer vs. winter	nd	1.7-fold increase	1.6-fold increase	Malanga et al., 2007
*Mya arenaria*	Fe 500 μM	4-fold increase day 0–17	nd	4-fold decrease day 0–17	González et al., 2010
**Mammals**					
Fetus rat brain	γ radiation 2 h	No change	No change	6-fold increase	Gisone et al., 2003
	γ radiation 4 h	Increase 51%	nd	No change	Gisone et al., 2003
Rat liver	Fe 500 mg/kg	2.7-fold increase	nd	No change	Galleano and Puntarulo, 1992; Rousseau et al., 2011

nd stands for non-determined.

To analyze the role of NO during senescence, Jasid et al. (2009) sprayed daily soybean cotyledons with an NO donor solution (sodium nitroprusside, SNP), from day 5 to 25. Differently from naturally senescent cotyledons, where NO content reached a maximum at day 10 of seedling development and declined, in SNP-treated cotyledons NO content was higher, and remained unchanged from day 10 to 25 after germination, as compared to control values. While naturally senescent cotyledons experimented no change in the content of LR^•^ during the studied period, SNP-treated cotyledons showed a decrease as time progressed (Table 1).

Shi et al. (2005) suggested that the protective effect of NO on the oxidative damage of thylakoid membrane proteins in *Phaseolus vulgaris* beans under UV-B radiation may be mediated by increasing the level of expression of genes encoding ROS-scavenging enzymes. Moreover, Jasid et al. (2006) showed that isolated chloroplasts from soybean leaves exposed to 2 μM NO for 30 min, decreased the rate of LR^•^ generation (Table 1). Also, Jasid et al. (2008) reported that axes from sorghum seeds incubated 24 h in the presence of SNP, showed a significantly higher NO content as compared to control axes, and LR^•^ content evaluated in the microsomal fractions was significantly lower, as compared to control membranes (Table 1).

## Lipid peroxidation and no in non-photosynthetic organisms

Data in Table 1 show that seasonality affects oxidative metabolism in digestive glands (DG) from *Nacella magellanica* limpets isolated from the Beagle Channel, Tierra del Fuego, Argentina. A significant increase in both LR^•^ and NO content in summer, as compared to winter, was reported (Malanga et al., 2007). However, studies on toxicological effects of Fe exposure under laboratory conditions showed that significant increases in lipid peroxidation were temporarily associated to decreases in NO content in DG from the bivalve *Mya arenaria* after 17 days of treatment (González et al., 2010) (Table 1).

*In vivo* γ irradiation of rat fetuses did not significantly affect neither the content of LR^•^ nor the content of TBARS in the brain up to 2 h post-irradiation (pi). However, 4 h after the exposure, a significant increase in the TBARS content was measured. These results are consistent with the hypothesis that changes could be produced in the brain at the early stages after exposure to γ radiation to limit free radical-dependent damage, since increased lipid peroxidation was only detected after 4 h pi. Gisone et al. (2003) showed that total NO synthase activity was increased after 30 and 60 min pi, and returned to control values after 2 h pi, and accordingly NO content was significantly increased (Table 1).

Galleano and Puntarulo (1992) showed that liver homogenates from Fe-dextran overloaded male Wistar rats showed a significant increase in TBARS 6 h post-injection, as compared to control rats (Table 1). Later, Galleano et al. (2001) pointed out that the significant increase in NO, assayed as DETC_2_–Fe–NO adducts 5 h after Fe administration could be an artifact due to the excess of Fe during the measurement. Recently, Rousseau et al. (2011) showed that one of the molecular footprints left by the reactions of ROS with biomolecules, the level of protein 3-nitrotyrosines, was not increased by Fe-dextran administration, suggesting that Fe overload in liver did not change NO cellular content (Table 1).

## Concluding remarks

The results summarized here implied the existence of a very complex regulatory interplay between NO and ROS. The multiple effects of NO on the process of lipid peroxidation imply that the net result will depend on the balance of competing factors. The rate and location of NO formation, and also the rate of formation of O^−^_2_, or other mitigating factors, will all contribute to the degree and the nature of the effect on lipid oxidation in a particular system. Detailed analysis of the molecular mechanisms in each condition is required. In this regard, no yet deeply studied NO reactions, such as NO binding to Fe and endogenous thiols and other nitrosyl–Fe complexes that seems to favor Fe release from the cell avoiding its accumulation, could reveal to be a key factor in NO cellular interactions and should be further characterized.

## References

[B1] Beligni M. V. Lamattina L. (2002). Nitric oxide interferes with plant photo-oxidative stress by detoxifying reactive oxygen species. Plant Cell Environ. 25, 737–748 10.1046/j.1365-3040.2002.00857.x

[B2] Estevez M. S. Malanga G. Puntarulo S. (2001). Iron-dependent oxidative stress in *Chlorella vulgaris*. Plant Sci. 161, 9–17 10.1016/S0168-9452(01)00364-8

[B3] Estévez S. Puntarulo S. (2005). Nitric oxide generation upon growth of Antarctic *Chlorella sp*. cells. Physiol. Plantarum 125, 192–201 10.1111/j.1399-3054.2005.00561.x

[B4] Galleano M. Aimo L. Borroni M. V. Puntarulo S. (2001). Nitric oxide and iron overload. Limitations or ESR detection by DETC. Toxicology 167, 199–205 10.1016/S0300-483X(01)00474-7 11578799

[B5] Galleano M. Puntarulo S. (1992). Hepatic chemiluminescence and lipid peroxidation in mild iron overload. Toxicology 76, 27–38 10.1016/0300-483X(92)90015-7 1475793

[B6] Gisone P. Boveris A. D. Dubner D. Perez M. R. Robello E. Puntarulo S. (2003). Early neuroprotective effect of nitric oxide in developing rat brain irradiated *in utero*. Neurotoxicology 24, 245–253 10.1016/S0161-813X(02)00166-3 12606296

[B7] Gisone P. Dubner D. Pérez M. R. Michelin S. Puntarulo S. (2004). The role of nitric oxide in the radiation-induced effects in the developing brain. In Vivo 18, 281–292 15341184

[B8] González P. M. Abele D. Puntarulo S. (2010). Exposure to excess dissolved iron *in vivo* affects oxidative status in the bivalve *Mya arenaria*. Comp. Biochem. Physiol. C 152, 167–174 10.1016/j.cbpc.2010.04.006 20398794

[B9] Jasid S. Galatro A. Villordo J. J. Puntarulo S. Simontacchi M. (2009). Role of nitric oxide in soybean cotyledon senescence. Plant Sci. 176, 662–668 10.1016/j.plantsci.2009.02.007

[B10] Jasid S. Simontacchi M. Bartoli C. G. Puntarulo S. (2006). Chloroplasts as a nitric oxide cellular source. Effect of reactive nitrogen species on chloroplastic lipids and proteins. Plant Physiol. 142, 1246–1255 10.1104/pp.106.086918 16980561 PMC1630751

[B11] Jasid S. Simontacchi M. Puntarulo S. (2008). Exposure to nitric oxide protects against oxidative damage but increases the labile iron pool in sorghum embryonic axes. J. Exp. Bot. 59, 3953–3962 10.1093/jxb/ern235 18832188 PMC2576640

[B12] Malanga G. Estevez S. Calvo J. Abele D. Puntarulo S. (2007). Seasonality effect on oxidative metabolism in *Nacella (P.) magellanica*. Comp. Biochem. Physiol. A 146, 551–558 10.1016/j.cbpa.2006.01.029 16516513

[B13] Malanga G. Puntarulo S. (1995). Oxidative stress and antioxidant content in *Chlorella vulgaris* after exposure to ultraviolet-B radiation. Physiol. Plantarum 94, 672–679 10.1111/j.1399-3054.1995.tb00983.x

[B14] Malanga G. Puntarulo S. (2012). The use of electron paramagnetic resonance (EPR) in the study of oxidative damage to lipids in aquatic systems, in Book on Oxidative Stress in Aquatic Ecosystems, eds Abele D. Zenteno-Savín T. Vázquez-Medina J. P. (Oxford, UK: Willey-Blackwell), 448–457

[B15] Qian H. Chen W. Li J. Wang J. Zhou Z. Liu W. (2009). The effect of exogenous nitric oxide on alleviating herbicide damage in *Chlorella vulgaris*. Aquat. Toxicol. 92, 250–257 10.1016/j.aquatox.2009.02.008 19297032

[B16] Rousseau I. Galleano M. Puntarulo S. (2011). Fe allocation in liver during early stages of endotoxemia in Fe-overload rats. Toxicol. Pathol. 39, 1075–1083 10.1177/0192623311425057 22006285

[B17] Sharpe M. A. Robb S. J. Clark J. B. (2003). Nitric oxide and Fenton/Haber–Weiss chemistry: nitric oxide is a potent antioxidant at physiological concentrations. J. Neurochem. 87, 386–394 10.1046/j.1471-4159.2003.02001.x 14511116

[B18] Shi S. Wang G. Wang Y. Zhang L. Zhang L. (2005). Protective effect of nitric oxide against oxidative stress under ultraviolet-B radiation. Nitric Oxide 13, 1–9 10.1016/j.niox.2005.04.006 15908241

[B18a] Simontacchi M. Buet A. Puntarulo S. (2011). The use of electron paramagnetic resonance (EPR) in the study of oxidative damage to lipids in plants, in Lipid Peroxidation: Biological Implications, ed Catalá A. (Kerala, India: Res. Signpost Transworld Res. Network), 141–160 ISBN: 978-81-7895-527-8

[B19] Simontachi M. Jasid S. Puntarulo S. (2004). Nitric oxide generation during early germination of sorghum seeds. Plant Sci. 167, 839–847 10.1016/j.plantsci.2004.05.028

[B20] Wink D. A. Cook J. A. Pacelli R. Degraff W. Gamson J. Liebmann J. (1996). The effect of various nitric oxide-donor agents on hydrogen peroxide-mediated toxicity: a direct correlation between nitric oxide formation and protection. Arch. Biochem. Biophys. 331, 241–248 10.1006/abbi.1996.0304 8660704

[B21] Wink D. A. Cook J. A. Pacelli R. Liebmann J. Krishna M. C. Mitchell J. B. (1995). Nitric oxide (NO) protects against cellular damage by reactive oxygen species. Toxicol. Lett. 82/83, 221–226 10.1016/0378-4274(95)03557-5 8597056

[B22] Yalowich J. C. Conery A. Thampatty P. Allan W. P. Baskaran R. Bhalla K. N. (1999). Sensitization of BCR-ABL^+^ cells to etoposide (VP-16) induced apoptosis: use of the tyrosine kinase inhibitor CGP57148B (Abstract). Proc. Am. Assoc. Cancer Res. 40, 4866

